# The variation of cloud amount and light rainy days under heavy pollution over South China during 1960–2009

**DOI:** 10.1007/s11356-017-0510-4

**Published:** 2017-11-09

**Authors:** Chuanbo Fu, Li Dan

**Affiliations:** 1 Hainan Meteorological Observatory, Haikou, 570203 China; 2 Key Laboratory of Regional Climate-Environment Research for Temperate East Asia, Institute of Atmospheric Physics, Chinese Academy of Sciences, Beijing, 100029 China

**Keywords:** Cloud amount, Light precipitation, Aerosol, AOD, Visibility, Sunshine duration

## Abstract

The ground observation data was used to analyze the variation of cloud amount and light precipitation over South China during 1960–2009. The total cloud cover (TCC) decreases in this period, whereas the low cloud cover (LCC) shows the obvious opposite change with increasing trends. LCP defined as low cloud cover/total cloud cover has increased, and small rainy days (< 10 mm day^−1^) decreased significantly (passing 0.001 significance level) during the past 50 years, which is attributed to the enhanced levels of air pollution in the form of anthropogenic aerosols. The horizontal visibility and sunshine duration are used to depict the anthropogenic aerosol loading. When horizontal visibility declines to 20 km or sunshine duration decreases to 5 h per day, LCC increases 52% or more and LCP increases significantly. The correlation coefficients between LCC and horizontal visibility or sunshine duration are − 0.533 and − 0.927, and the values between LCP and horizontal visibility or sunshine duration are − 0.849 and − 0.641, which pass 0.001 significance level. The results indicated that aerosols likely impacted the long-term trend of cloud amount and light precipitation over South China.

## Introduction

Interaction between aerosol, cloud, and precipitation has become one of the frontiers in the atmospheric science (Lohmann and Lesins 2002; Penner et al. 2004). Aerosols affect cloud microphysical process and precipitation process mainly through the direct and indirect ways (Ramanathan et al. 2001). Direct effect is detailed as aerosol particle scattering or absorbing solar radiation, and it changes the net absorption of solar radiation flux of land-air system, then impacts surface radiation balance. Indirect effect can be divided into a Twomey effect (Twomey 1959; Twomey and Wojciechowski 1969; Twomey 1974) (first indirect effect) and Albrecht effect (Albrecht 1989; Novakov and Penner 1993) (second indirect effect), and it is referred as aerosol particles to impact cloud water content, cloud droplet radius, and cloud lifetime through cloud condensation nuclei (CCN). Actually, light precipitation was mainly inhibited by aerosols through the indirect effect (Qian et al. 2009a; Qian et al. 2009b). High concentration of aerosol particles in the cloud retards the process of warm rain effectively. The increased CCN weakens the mixed phase stratocumulus, and the cloud life is extended and cumulative rainfall decreases. Light precipitation mainly belongs to low cloud precipitation, whereas low cloud precipitation forms during warm rain process, so there exists an intimate linkage among aerosol particles, low cloud cover (LCC) and light precipitation (Liu et al. 2011; Wu et al. 2017).

**Fig. 1:**
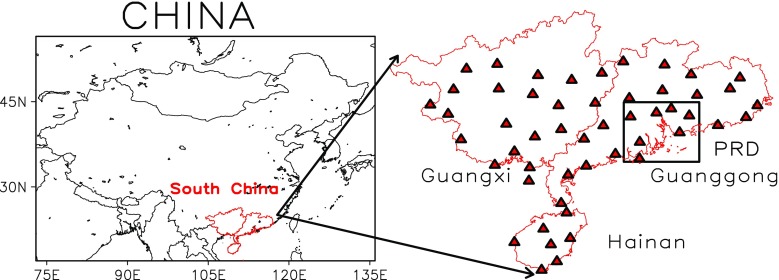
The location of 51 weather stations in South China, rectangle standing for the range of Pearl River Delta (PRD)

The main method currently used to study the aerosol effect on cloud and precipitation depends on the satellite data (Ferek et al. 1998; Rosenfeld 2000; Heymsfield and McFarquhar 2001; Yum and Hudson 2002), model simulation (Menon et al. 2002; Giorgi et al. 2003; Ackerman et al. 2004; Takemura et al. 2005), and surface observation data (Feingold et al. 1999; Khain et al. 1999; Saleeby and Cotton 2004; Teller and Levin 2006). Yin and Chen (2007) used a dynamic cloud model with detailed microphysics of both warm and ice phase processes, and found that dust aerosol could transform to cloud condensation nucleus (CCN) and ice nucleus (IN), thus affected cloud and precipitation. Huang et al. (2007) developed a regional coupled climate-chemistry-aerosol model to investigate the anthropogenic aerosol effect on regional precipitation in East Asia. Zhao et al. (2006) found that precipitation in Eastern China reduced significantly for nearly 40 years, and it can be attributed to the increase of aerosol particles concentration. Choi et al. (2008) found that aerosol diurnal variation has positive correlation with moderate precipitation frequency, and has negative correlation with light precipitation frequency, thus aerosol concentration increased while precipitation and humidity in summer decreased year by year. Consequently, it is necessary to explore the mechanism between aerosols, LCC and light precipitation (Ma et al. 2015). However, due to the lack of observational aerosol data, the research on aerosol cloud physics as well as climate effect is rare in previous studies. South China is one of the fastest-growing economy regions in recent decades, especially in the Pearl River Delta (PRD, Fig. 1). The air pollution is quite serious and has aroused widespread concern in the government and the public (Wu 2005; Wu et al. 2009). Thus, the aim of this study is to explore the impact on light precipitation and cloud amount by aerosol particles over South China in the past 50 years.

## Data and method

### Data

The daily datasets used here were obtained from the China Meteorological Administration (CMA), which included total cloud cover (TCC), LCC, daily precipitation, visibility, and sunshine duration from January 1960 to December 2009. The stations with data more than 5% missing for a year were filtered out, and only data subject to strict quality control were used for 57 stations (shown in Fig. 1). In addition, aerosol optical depth (AOD) at 550 nm retrieved from MODIS (moderate-resolution imaging spectroradiometer) satellite during 2001 to 2009 was also adopted, and the resolution of AOD is 1° × 1°.

### Climatic trend coefficient and the classification

Climatic trend coefficient (*r*_*xt*_) was calculated by Formula (1), which is defined as a correlation coefficient between elements of *n* (year) time sequence and natural series as 1, 2, 3…, *n*:

1 $$ {r}_{xt}=\frac{\sum \limits_{i=1}^n\left({x}_i-\overline{x}\right)\left(i-\overline{t}\right)}{\sqrt{\sum \limits_{i=1}^n{\left({x}_i-\overline{x}\right)}^2\sum \limits_{i=1}^n{\left(i-\overline{t}\right)}^2}} $$

where *n* is the time by years, *x*_*i*_ stands for an element magnitude in the *ith* year, $\overline{x}$ represents the average sample variable, and $\overline{t}=\left(n+1\right)/2$. *r*_*xt*_ is the standardized linear regression coefficient, which has eliminated the influence of numerical value by mean square error and unit. In addition, statistic methods of regression analysis, tendency fit, and correlation analysis (Wei 2007) were used in this study.

## Result analysis

### Annual mean trends of cloud amount and small rainy days

**Fig. 2:**
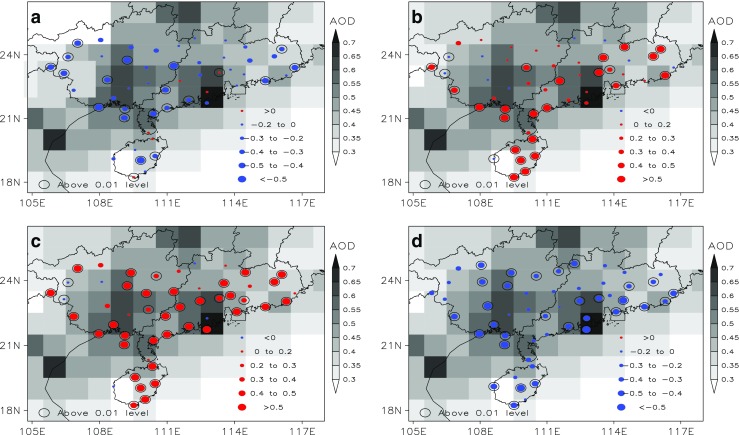
Spatial distribution of AOD and climatic trend coefficients of total cloud cover (**a**), low cloud cover (**b**), LCP (**c**), and small rainy days (**d**) over SC

Figure 2a and b shows the spatial distribution of AOD and climatic trend coefficients of TCC and LCC over SC. It shows high AOD over SC, with two obvious maximum centers above 0.6, which are located in south Guangxi and PRD region, respectively. The value in PRD region can be up to 0.7, where many cities cluster with million residents such as Guangzhou, Hong Kong, Shenzhen, Dongguan, Foshan, and Zhuhai. The enhanced levels of anthropogenic activities and population can lead to an excessive emission of atmospheric aerosol (Wu 2005; Wu et al. 2009). From Fig. 2a, there is an obvious decline of TCC in South China and 33.3% stations exceed the 0.01 significance level, with the exception of a few stations in PRD regions and Hainan province. The climatic trend coefficients exceed 0.01 significance level can be found in stations of west and east Guangxi, east Hainan, and coastal region of Guangdong. By contrast, most stations show a significant increasing trend of LCC and 44.4% exceed 0.01 significance level, especially in the coastal region of Guangdong and Guangxi, east Hainan, where the climatic trend coefficients are larger than 0.5.

In this section we also analyzed the climatic trend coefficients of Low Cloud Percentage (defined as low cloud cover/total cloud cover, LCP) and small rainy days (defined as 0.1 ≤ *P* ≤ 10 mm day^−1^), which is shown in Fig. 2c and d. The obvious increase in LCP can be found in nearly all stations and 78.9% of them pass 0.01 significance level. Four stations show a slight declined trend for LCP and three of them located in west of Guangxi and one in PRD region. For small rainy days, all the stations showing a decreasing trend, and 52.6% of them pass the 0.01 significance level. Obviously, this phenomenon leads to the questions that why LCC had an increase trend when TCC was declining during the past 50 years? Do aerosols impact cloud amount and light precipitation over South China? Actually, the possible reason for these questions might be attributed to the anthropogenic pollution. The aerosols change the microphysical characteristics of cloud through influencing the microphysical process, then effect on precipitation processes (Ramanathan et al. 2001). The dramatic increase in pollutant emissions can result in a significant increase of anthropogenic aerosols and relevant secondary aerosols. The enhanced aerosols in the atmosphere might further increase LCC and reduce the small rainy days through the Twomey effect and Albrecht effect (Warren et al. 2007; Sun et al. 2014; Zhang et al. 2015).

**Fig. 3:**
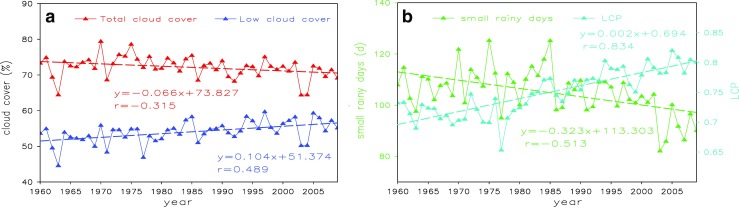
Time series of total cloud cover, low cloud cover, LCP, and small rainy days during 1960–2009 averaged over SC stations

Figure 3a and b shows time series of TCC, LCC, small rainy days, and LCP. It suggests that LCC increases markedly in contrast with TCC over South China, and the discrepancy between them was reducing over this period. In addition, the regression coefficient of LCC is 1.015% per 10 years and its climatic trend coefficient is 0.489 (Table 1), exceeding the 0.001 significance level. Small rainy days continuously decline from 1960 to 2009, with two highest values in 1975 and 1985, which is consistent with the result of Qian et al. (2009a, 2009b). On the contrary, a significant increasing trend of 0.021 per 10 years is also shown for LCP averaged over 57 stations in South China. It can be found that the trend of LCP correlated well with small rainy days negatively. Climatic trend coefficients of LCP and small rainy days are 0.834 and − 0.513, respectively. Both of them also passed the 0.001 significance level.

**Table 1:** Statistics of annual mean cloud amount and small rainy days over South China

	Mean	SD	CTC	RC	*P*
Total cloud cover	72.152%	3.013%	− 0.315	− 0.643·10a^−1^	0.05
Low cloud cover	54.016%	3.06%	0.489	1.015·10a^−1^	0.001
LCP	0.749	0.038	0.834	0.021·10a^−1^	0.001
Small rainy days	105.06 day	9.09 day	− 0.513	− 3.168 day·10a^−1^	0.001

*SD*, standard deviation; *TC*, climatic trend coefficient; *RC*, regression coefficient; *P*, the level of significance

### Annual mean trends of total precipitation

**Fig. 4:**
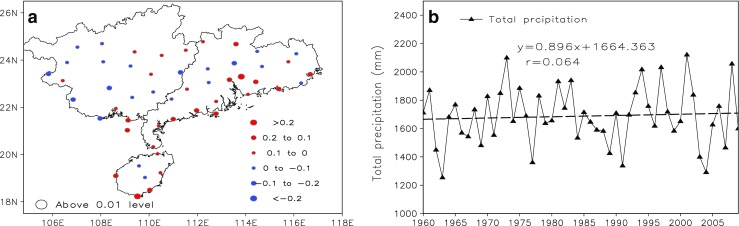
Spatial distribution of climatic trend coefficients of annual precipitation over SC (**a**) and its time series (**b**) averaged over SC during 1960 to 2009. Stations whose trends are significant at the 95% confidence level are circled in black

The trend of cloud amount and light precipitation is mainly affected by natural factors such as atmospheric structure and water vapor content, and human factors like aerosol particles. Therefore, it is appropriate to first discuss the atmospheric circulation patterns over South China. Actually, similar studies have previously shown an increasing trend of precipitation over South China and a decreasing trend over North China, and this pattern of change has been called the “south wet/north drought” phenomenon (Gong and Ho 2002; Hu et al. 2003). As shown in the spatial distributions of the climatic trend coefficients of annual precipitation over South China (Fig. 4a), we can find that 50.9% stations show a slight increasing trend and the others show a weak decreasing trend of annual precipitation during the past 50 years, not exceeding 0.01 significance level. Figure 4b presents the time series of annual precipitation averaged over South China, the increasing trend of annual precipitation is very weak too, and the climatic trend coefficient and regression coefficient are 0.064 and 8.96 mm·10a^−1^, respectively, not passing 0.01 significance level. From the above analysis, it is suggested that annual precipitation over South China did not change much under the large-scale background of atmospheric circulation change in the past 50 years. Therefore, the possible reason for the trend of cloud amount and light precipitation can attribute to human factor (aerosol particles), not to natural factor, which we investigated based on further data analysis.

### Annual mean trends of horizontal visibility and sunshine duration

**Fig. 5:**
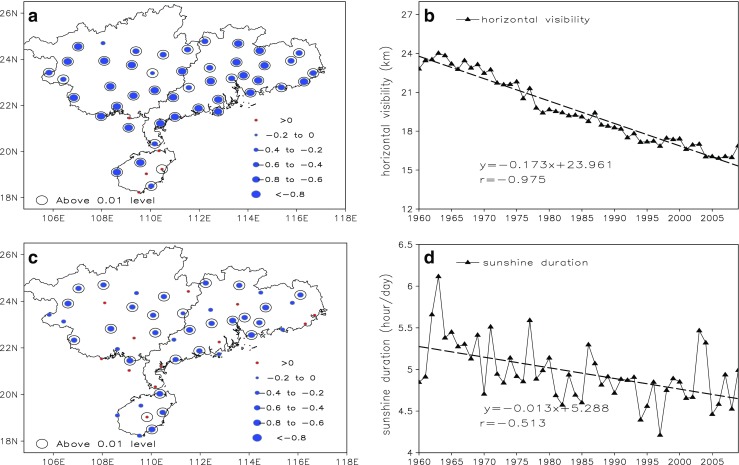
Spatial distribution of climatic trend coefficients of horizontal visibility (**a**) and sunshine duration (**c**) over SC and their time series (**b** and **d**) averaged over SC during 1960 to 2009. Stations whose trends are significant at the 95% confidence level are circled in black

According to the above analysis, it can be understood that LCP increased and small rainy days declined obvious during the past 50 years, which is mainly due to aggravated anthropogenic emission of aerosol particles. To further investigate the impact of human activities on cloud amount and light precipitation during 1960 to 2009, we discuss anthropogenic emission data from 1960 to 2009 over South China to explore the long-term trend and variability of aerosol particle characteristics. However, we are not able to calculate the long-term trend for AOD because data is not available for the earlier period of the 1960s–2000s, but we have more than 50 years of proxy data of sunshine duration and visual range. Figure 5a and c shows Spatial distribution of climatic trend coefficients of horizontal visibility and sunshine duration over SC. Horizontal visibility decreased across the whole of South China and 91.2% stations passed the 0.05 significance level. Especially in the coastal region and the PRD region, the climatic trend coefficient can be less than − 0.8, and the reason is likely related to the increased emissions of aerosol particles in South China under the backdrop of rapid economic development, which can lead to gray-haze weather and cause a reduction in visibility. Similarly, Sunshine duration shows a significant decline in most stations over South China and 45.6% stations passed the 0.05 significance level during the past 50 years. Figure 5b and d shows time series of horizontal visibility and sunshine duration averaged over South China during 1960 to 2009. Both horizontal visibility and sunshine duration show an obvious decline during the past 50 years; regression coefficients of horizontal visibility and sunshine duration are − 1.37 km·10a^−1^ and − 0.13 h day^−1^·10a^−1^, and their climatic trend coefficients are − 0.975 and − 0.513, respectively, passed the 0.001 significance level. Similar to the conclusion of Fu and Dan (2014), the reason can be attributed to excessive emissions of contaminants in South China.

### Relationship between cloud amount and anthropogenic air pollutants

**Fig. 6:**
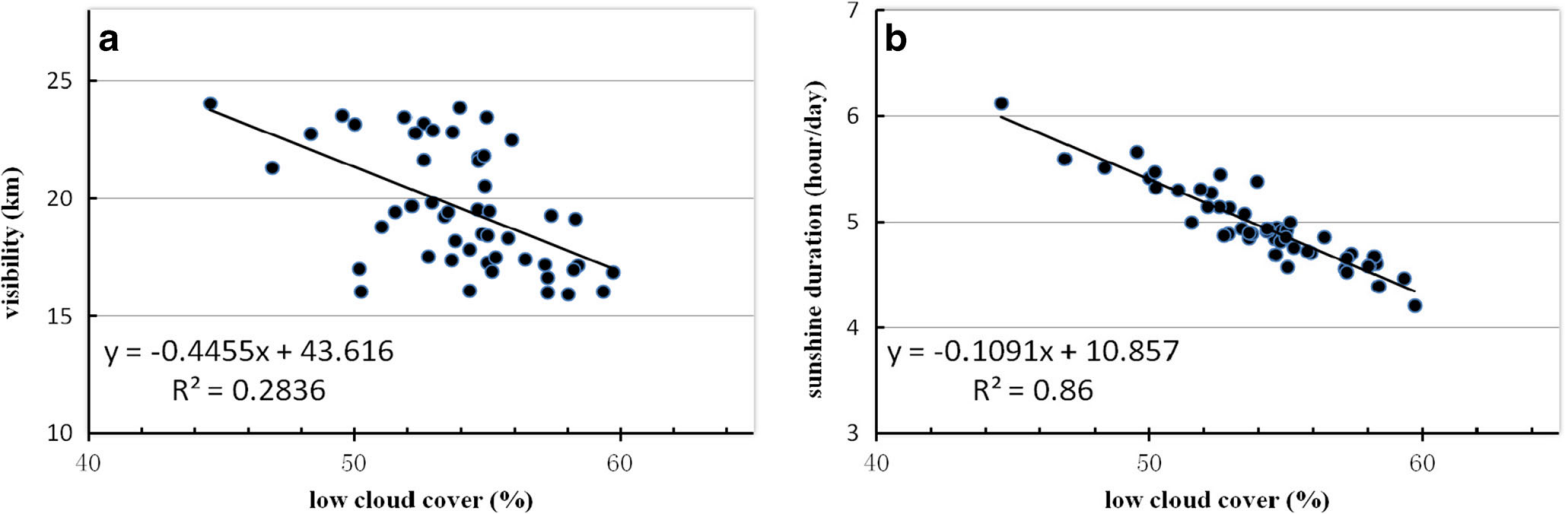
The scatter plot of low cloud cover and horizontal visibility (**a**) and sunshine duration (**b**) during 1960–2009 at weather stations over South China

**Fig. 7:**
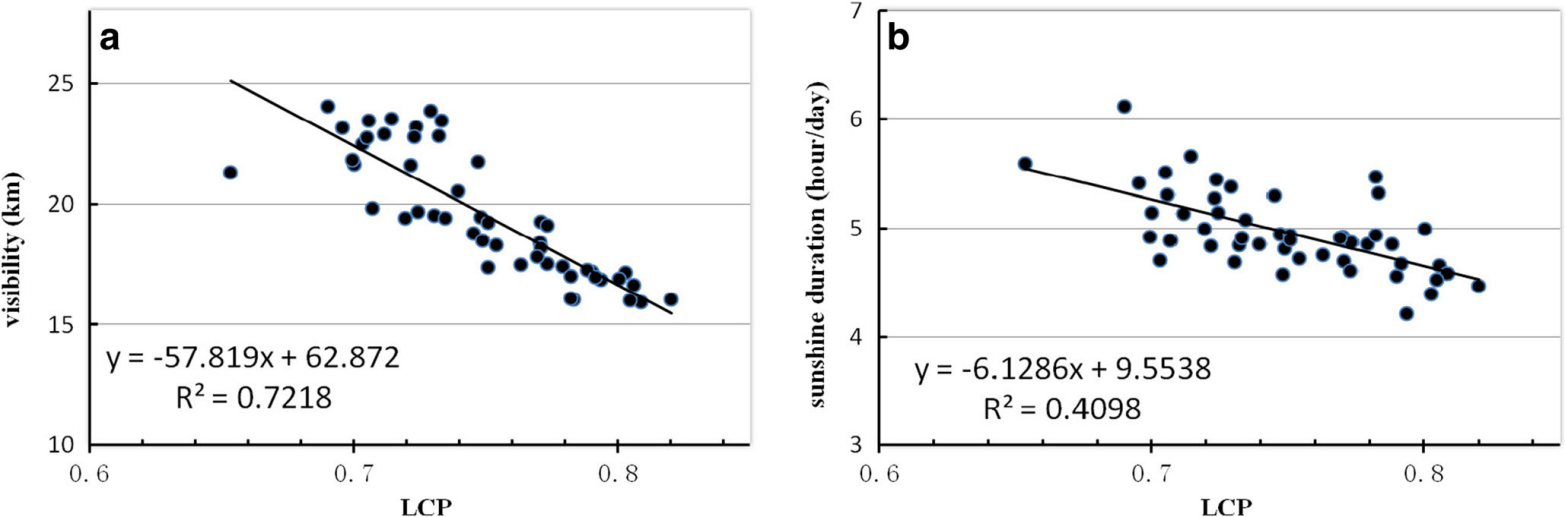
The same as Fig. 6, but for LCP

To further investigate the relationship between cloud amount and anthropogenic air pollutants, 50 years of data for horizontal visibility and sunshine duration were used as a substitute for the short-term availability of AOD data. As shown in Fig. 6a and b, when horizontal visibility decreases or sunshine duration decreases, the LCC increase. In Fig. 6a, it is obvious that when horizontal visibility is below 20 km, the LCC increases to 52% or more. Similarly, in Fig. 6b, while sunshine duration decreases to 5 h day^−1^ or less, LCC are mostly above 52%. The correlation coefficients of horizontal visibility and sunshine duration with LCC are − 0.533 and − 0.927, respectively, passed the 0.001 significance level. As shown in Fig. 7a and b for LCP, when horizontal visibility decreases from 20 to 15 km or sunshine duration decreases from 5 to 4 h·day^−1^, LCP increases significantly. There is also a strong relationship between LCP and horizontal visibility or sunshine duration, their correlation coefficients are − 0.849 and − 0.641, respectively, which all passed the 0.001 significance level. The increasing trends in LCC and LCP are consistent with the conclusion above that the influence of aerosols would mainly result in low cloud amount increase, then restrain light precipitation.

Based on the above analysis, we can infer that the increased emissions of aerosol particles in South China during the past 50 years have an impact upon low cloud amount through influencing the microphysical process, then effect on light precipitation processes. But how these mechanisms might happen? Actually, we know that aerosols can decrease the amount of solar radiation that reaches the land surface, and therefore cause less heat to be available for evaporation and energizing convective rain clouds (Ramanathan et al. 2001). The fraction of radiation that is not reflected back to space by the aerosols is absorbed into the atmosphere, leading to heating of the air above the surface. This stabilizes the lower atmosphere and suppresses the generation of convective clouds (Koren et al. 2008). In addition, light precipitation over South China is mainly caused by convective clouds (Rosenfeld et al. 2008). Accompanied with aggravated anthropogenic emission of aerosol particles in South China during the past 50 years, a higher concentration of CCN with smaller radii, leads to less cloud droplets being coalesced into precipitation, which in turn prolongs the existence of the low cloud through positive feedback, then suppressed light precipitation (Cheng et al. 2005). Therefore, to account of the above analysis and observations together, the implication is that anthropogenic aerosols are a principal factor in increasing LCC and reducing light precipitation in South China.

## Conclusions and discussions

It was argued that surface observations of cloud amount and light precipitation were influenced by the aerosol indirect effect over South China (Duan and Liu 2011). In this paper, we focus on analyzing the changes in cloud amount and light precipitation for the period 1960–2009. AOD, horizontal visibility, and sunshine duration are used to characterize the aerosol loading. The result shows that an obvious opposite change trends for TCC and LCC were found during the past 50 years over South China. LCP (low cloud cover/total cloud cover) have increased and small rainy days (< 10 mm day^−1^) decreased significantly (passing 0.001 significance level) for the period 1960–2009, and this phenomenon appears to be closely related to increasing levels of air pollution in the form of anthropogenic aerosols.

As the aerosol particle loading increases, both LCC and LCP increase while TCC and small rainy days decrease for the period 1960–2009. For LCC, when horizontal visibility declines to 20 km or sunshine duration decreases to 5 h day^−1^, LCC increases to 52% or more. For LCP, when horizontal visibility decreases from 20 to 15 km or sunshine duration decreases from 5 to 4 h·day^−1^, LCP increases significantly. In this period, LCC and LCP show a significant negative correlation with aerosol loading, the correlation coefficients between LCC and horizontal visibility or sunshine duration and are − 0.533 and − 0.927, and the values between LCP and horizontal visibility or sunshine duration are − 0.849 and − 0.641, which passed 0.001 significance level. Thus, we confirm that the excessive emissions of aerosol particles had an impact upon cloud amount and light precipitation obviously during the past 50 years over South China. This represents a serious environmental problem for the region that requires careful consideration. If it persists without any attempts of amelioration, it is likely to adversely affect the socioeconomic development of South China and be harmful to the sustainable development of China.
